# Knowledge Representation for Multi-Scale Physiology Route Modeling

**DOI:** 10.3389/fninf.2021.560050

**Published:** 2021-02-16

**Authors:** Natallia Kokash, Bernard de Bono

**Affiliations:** ^1^Peoples' Friendship University of Russia (RUDN University), Moscow, Russia; ^2^Auckland Bioengineering Institute, University of Auckland, Auckland, New Zealand

**Keywords:** physiology, multi-scale model, knowledge management, anatomy, connectivity, ontology

## Abstract

We present a framework for the topological and semantic assembly of multiscale physiology route maps. The framework, called ApiNATOMY, consists of a knowledge representation (KR) model and a set of knowledge management (KM) tools. Using examples of ApiNATOMY route maps, we present a KR format that is suitable for the analysis and visualization by KM tools. The conceptual KR model provides a simple method for physiology experts to capture process interactions among anatomical entities. In this paper, we outline the KR model, modeling format, and the KM procedures to translate concise abstraction-based specifications into fully instantiated models of physiology processes.

## 1. Introduction

Physiology process models take into account the anatomical routes of communication that are necessary for mechanisms to occur. For example, process models study mechanisms in which:

- an increase in atrial pressure gives rise to an increase in glomerular filtration rate;
- stomach filling is followed by colon emptying;
- low oxygen partial pressure in the alveolus leads to an increased red blood cell count.

ApiNATOMY provides a knowledge representation (KR), and knowledge management (KM) tools, for the *topological* and *semantic* modeling of process routes and associated anatomical compartments in multiscale physiology. The development of ApiNATOMY is a work-in-progress and is coupled to integrative efforts by communities in physiology and medicine [e.g., SPARC (National Institutes of Health), HuBMAP (Snyder et al., 2019)].

In this context, the requirements collected in previous work (de Bono et al., 2012, 2017) identified two core sets of KR organizing principles for physiology processes, namely:

1. A first set of organizing principles for flow processes is drawn from:
  (a) *Fick's Law*, which emphasizes the dependency of exchange between conduit compartments on the surface area and permeability of the interface between them;
  (b) *Poiseuille equation*, which links the resistance of a fluid conduit to its length and internal radius; and
  (c) *LaPlace's work*, which relates the pressure in the lumen of a conduit to the tension in the wall based on he internal radius and wall thickness.
2. A second set of organizing principles is gained from the prevalence of tree-like (i.e., branching) patterns in biological conduit architecture. Such arborizations have evolved to manage the gradual transition from:
  (a) thick-walled root-proximal vessels adapted for high-pressure high-velocity flow (e.g., aorta, trachea, ureter), needed for long range propulsion to
  (b) high-surface area thin-walled leaf-distal vessels that permit low-pressure low-velocity flow required for sustainable exchange (e.g., capillaries, alveoli, nephrons).
  Transfers between conduit systems (e.g., material transfer between blood and urine, or digested food and lymph) take place across adjacent conduit walls (i.e., transmural) of leaf-distal vessels.

The first set of principles suggests that describing conduits that broker flow processes should take into account radius, length, wall thickness and surface area of these compartments. The second set suggests that the representation of conduit arborizations (e.g., arterial tree, biliary tree, neuron), as well as the transmural transfer between such arborizations, should explicitly take into account branching patterns and wall-to-wall interactions between leaf-distal vessels.

Given the above requirements, the ApiNATOMY KR model should provide ways to model:

- *process edges* to depict energy and mass transactions, in particular, transmission, storage, and dissipation (de Bono et al., 2017);
- structural entities such as molecules, cells or tissue units (i.e., in Basic Formal Ontology (BFO) (Arp et al., 2015) parlance, an independent continuant). The construct responsible for this kind of modeling in the ApiNATOMY's KR is called *lyph*. A lyph can be used as container (i.e., as compartment), as component (i.e., as regional or constituent part), or as process participant (i.e., a conduit);
- *route* maps, or a combination of *lyphs* and *process edges* such that there is a one-to-one mechanistic correspondence between a process edge and a lyph that conveys, or brokers, the transaction represented by the edge. A route can, therefore, represent a chemical reaction brokered by an enzyme, the diffusive flow of an ion across a membrane, or the advective flow of fluid down an anatomical conduit. Concatenations of routes can be assembled to represent complex multiscale biological models.

Using exemplar route maps for context and illustration, this paper defines a *generic KR model for ApiNATOMY* that addresses representational requirements for routes in multi-scale physiology modeling. The KR schema for this model provides a contract between the input data used in route map construction and the *ApiNATOMY KM toolset* (Kokash) that validates and transforms the data into an expanded entity-relationship model. This model is manipulated by the *ApiNATOMY viewer* (Kokash), an open-source web tool that renders physiology models, and places visual artifacts depicting physiology entities into a force-directed 3D layout.

The paper is organized as follows: section 2 provides a high-level description of sample physiology models represented as route maps in ApiNATOMY. Section 3 introduces the ApiNATOMY conceptual schema. Section 4 describes model transformation steps to obtain fully instantiated entity-relationship object models from the initial template-based specifications. Section 5 focuses on model display. In section 6, we discuss the implications of this study.

## 2. Requirements and Use Cases

The mainstay of the ApiNATOMY is the notion of *lyph*. The high-level lyph features described here will be further considered in the context of the four exemplar ApiNATOMY models discussed below.

**Figure 1:**
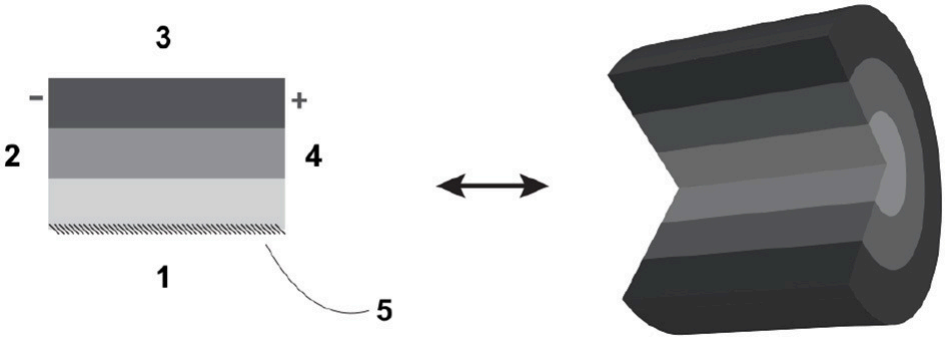
Definition of lyph: borders and axis of rotation.

**Figure 2:**
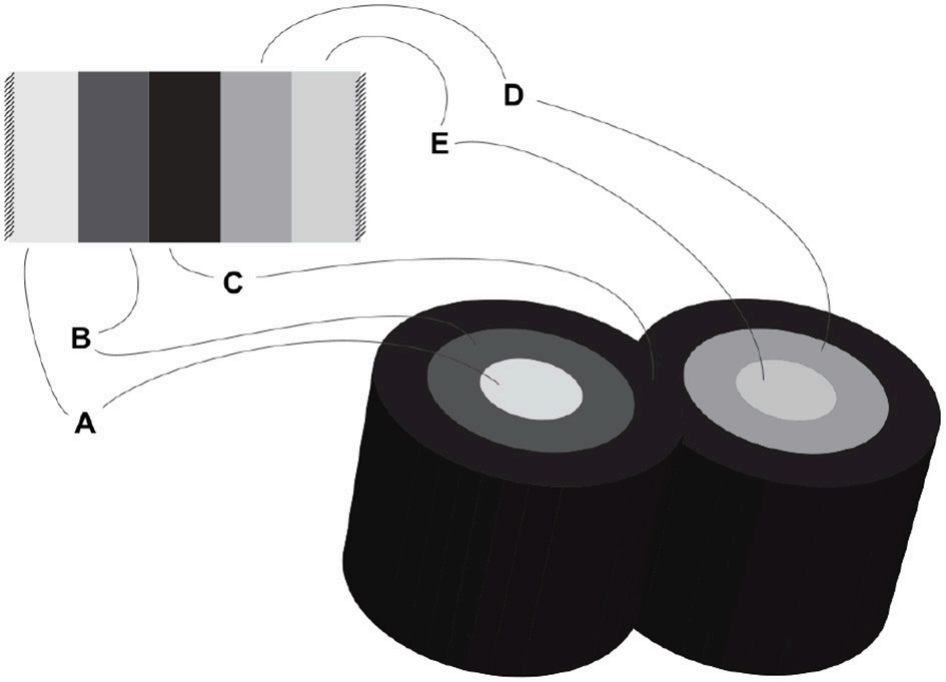
Coalescent assembly.

Lyph features are designed to manage knowledge about compartmental measurements, and related flow processes, including *annotation metadata* to reference ontologies, (e.g., declaring a lyph object as representing an instance of a biological structure class) and *mereotopological information*, such as:

- *Borders* and their polarity: given that for any lyph, two longitudinal borders are distinguished by dint of their proximity to the axis of rotation and two radial borders are arbitrarily labeled for distinction. In Figure 1, longitudinal borders are marked as 1 and 3, radial borders as 2 and 4, and the axis of rotation (in this case, coinciding with longitudinal border 1) is marked as 5. Lyphs can be annotated with *length* and *thickness* in the form of distributions of one-dimensional measurements of length along longitudinal and radial borders, respectively.
- *Composition* in terms of parts, that include:
  - regions as lyphs contained in another lyph, which may extend to *layers* (the lyph in Figure 1 has three layers): regions that run longitudinally from one radial border to another;
  - *constituent materials*, which are aggregates, including other lyphs, constituting the fabric of a lyph.
- *Configuration*: conduits for flow take one of three topological configurations: the Bag, which indicates a terminus for flow of material in its innermost layer; the Tube, which indicates continuation of flow of material in its innermost layer; and the Cyst, a complete envelopment of material within its innermost layer.
- *Assemblies*:
  - *Axial assembly* takes place along the axis of rotation by sharing radial borders. It allows lyphs to join into more complex constructions such as arborizations.
  - *Arborization assemblies* are axial assemblies imbued with branching probabilities which, in combination with length and thickness distributions, make for a parameterizable model for different kinds of anatomical trees.
  - *Coalescent assembly*, illustrated in Figure 2, occurs orthogonal to the axis of rotation by sharing longitudinal borders. It allows for conduits to link transmurally by sharing the outermost layer of their wall.

Application of lyph assemblies is discussed below.

**Figure 3:**
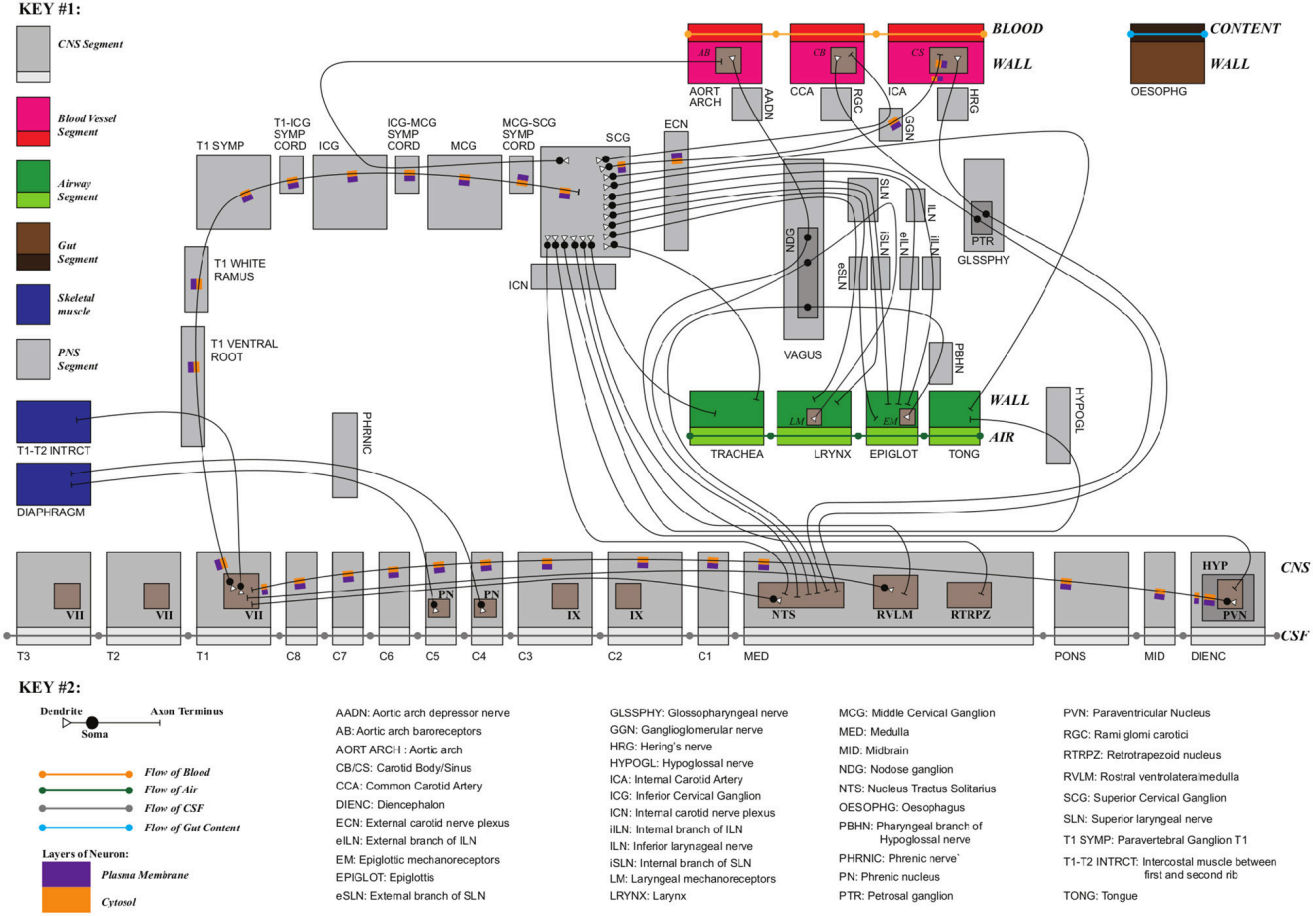
Neural circuits tracking arteries and airways in the neck.

**Figure 4:**
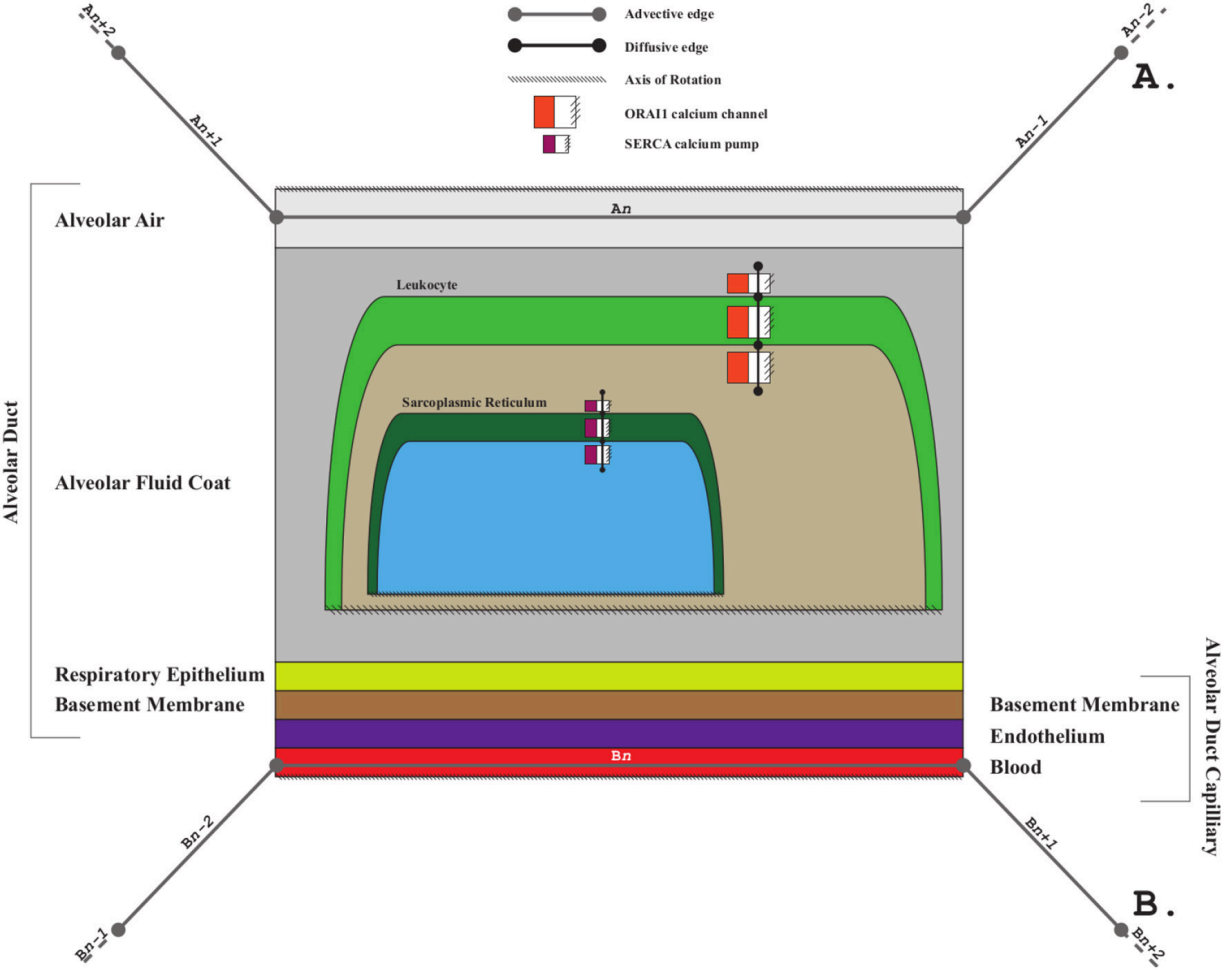
Leukocyte calcium handling in alveolar fluid.

**Figure 5:**
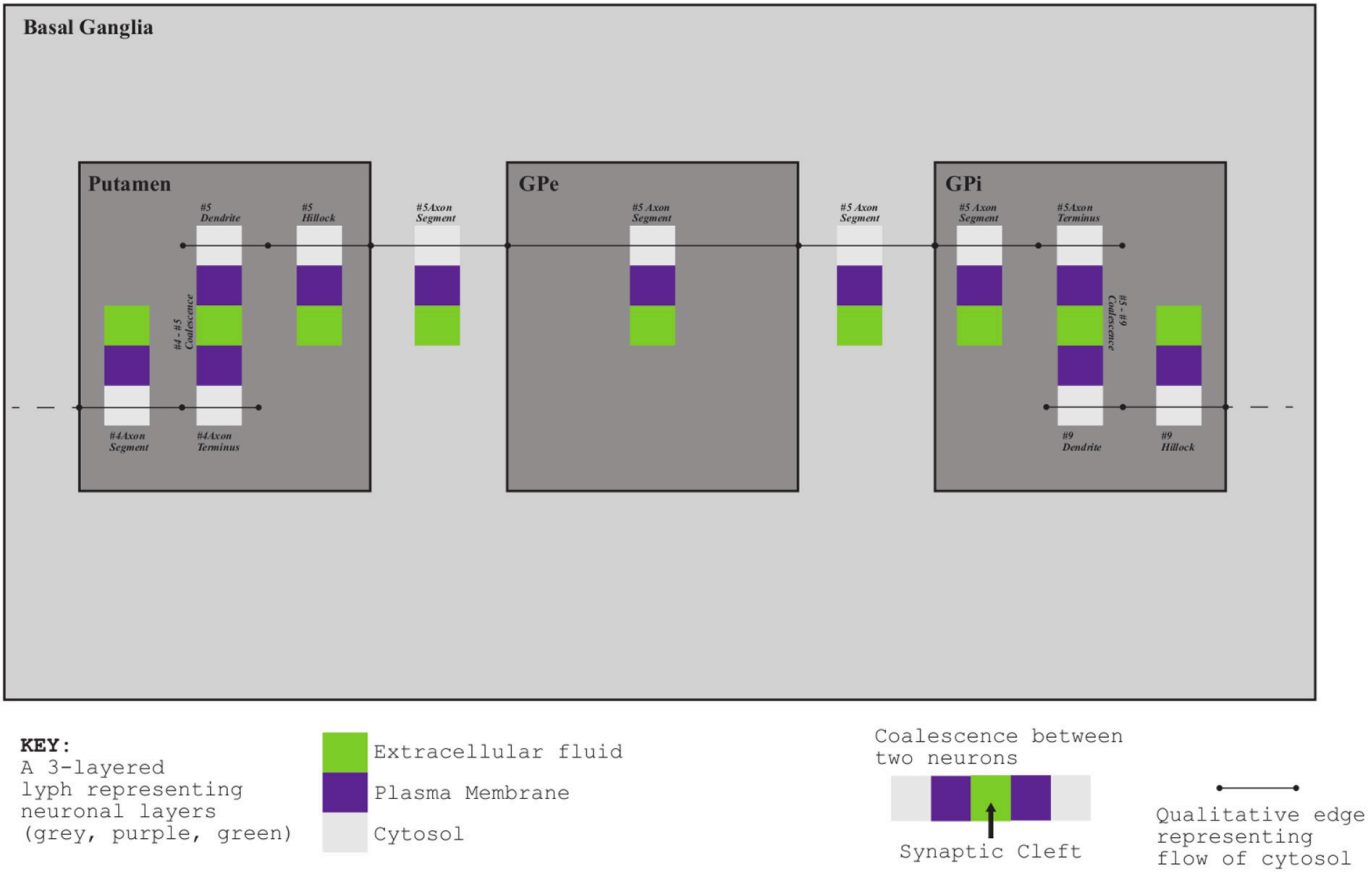
Basal Ganglia mock-up.

**Figure 6:**
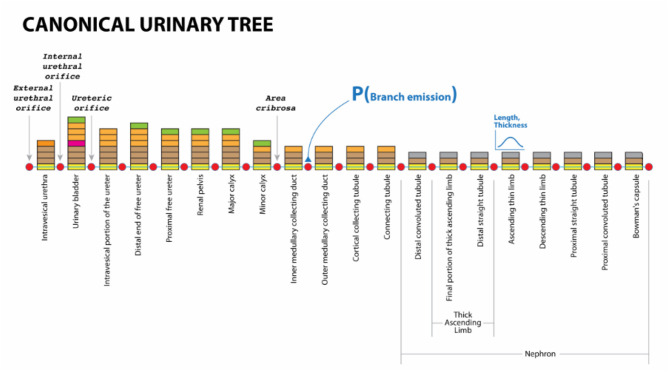
Urinary Omega Tree mock-up.

**Exemplar 1:** Figure 3 shows a schematic mock-up of an ApiNATOMY physiology route. The model features neural circuits tracking arteries and airways in the neck. In this model, four arborized conduit systems are represented as axial assemblies: cardiovascular (red, conveying flow of blood), airway (green, flow of air), central nervous (grey, cerebrospinal fluid), and neurons (shown as simple lines that represent the flow of neuronal cytosol).
  Each arborization system is modeled as a linear concatenation of process edges, each edge conveyed by a lyph that has, as its innermost layer, a representation of the fluid flowing through that system (e.g., blood). The other layers of the conveying lyph represent the wall of the conduit.
  Placing, or nesting, one lyph in another lyph provides the means to represent compartments as regions within compartments that house them (e.g., the Carotid Body, CB, brown square, shown housed by the Common Carotid Artery, CCA, a 2-layered red rectangle. Note the CB is placed in the outer layer, i.e., in the wall of the CCA). In situations where one arborized system is to be threaded along another arborized system, as in the case of threading neuron trees along the Central Nervous System (CNS) tree, the KM tools provide the means to automate the placement of conveying lyphs of one system (e.g., the lyphs representing segments of neurons) as regions of conveying lyphs of another system (e.g., the outer layer of the central nervous lyphs between the Diencephalon and T1 levels in the arborization).
**Exemplar 2:** The model shown in Figure 4 represents the compartmental layout relevant to communication between leaf-distal vessels. One of the lyph layers, labeled “Alveolar Fluid Coat,” has cells (known as Leukocytes) as one of its material constituents. The second material constituent is the ion Calcium (not shown in Figure 4).
  The two leaf-distal vessels in this model belong to two distinct arborizations. The process edge concatenation labeled *A* represents air flow in the airway arborization, where edge *A*_*n*_ is brokered by a 4-layer conveying lyph labeled “Alveolar Duct.” Concatenation *B* represents blood flow in the cardiovascular arborization, where edge *B*_*n*_ is brokered by 3-layer conveying lyph “Alveolar Duct Capillary.” The two conveying lyphs both have Basement Membrane as their outermost layer. This allows the two lyphs to engage on a 6-layer connecting coalescence by having the Basement Membrane layer in common.
  A 2-layer lyph of type Cyst represents the Leukocyte cell, and another 2-layer cyst models the Sarcoplasmic Reticulum compartment in the innermost layer of the Leukocyte cell. The route conveying Calcium material between the innermost layer of the Sarcoplasmic Reticulum and the outside of the Leukocyte is brokered by two axial assemblies: one representing the SERCA calcium pump, and the other the ORAI1 calcium channel.
**Exemplar 3:** The Basal Ganglia (BG) are a group of subcortical nuclei at the base of the forebrain and above the midbrain. Figure 5 shows the schematic representation of BG interconnections. In this figure, the neuron that courses through the GPe consists of two arborizations, such that leaf-distal coalescences with other neurons represent synapses in the Putamen and GPi.
**Exemplar 4:** Figure 6 shows a mock-up of an arborization representing the structure of a urinary system from Urethra to Nephron. In human, this model branches to create one Bladder, two Ureters, about 24 Minor Calyces and circa 2 million Nephrons. In more extensive ApiNATOMY models, the Nephron engages in multiple coalescences with Blood Vessel leaf-distal lyphs.

We will use the aforementioned mock-ups to validate the usability of the ApiNATOMY KM.

## 3. Model Definition

In constructing route models, the ApiNATOMY KM tools work with input data specified by users. This data should conform to the framework's conceptual schema that provides a contract between the user-defined data and the input format expected by the model viewer.

**Figure 7:**
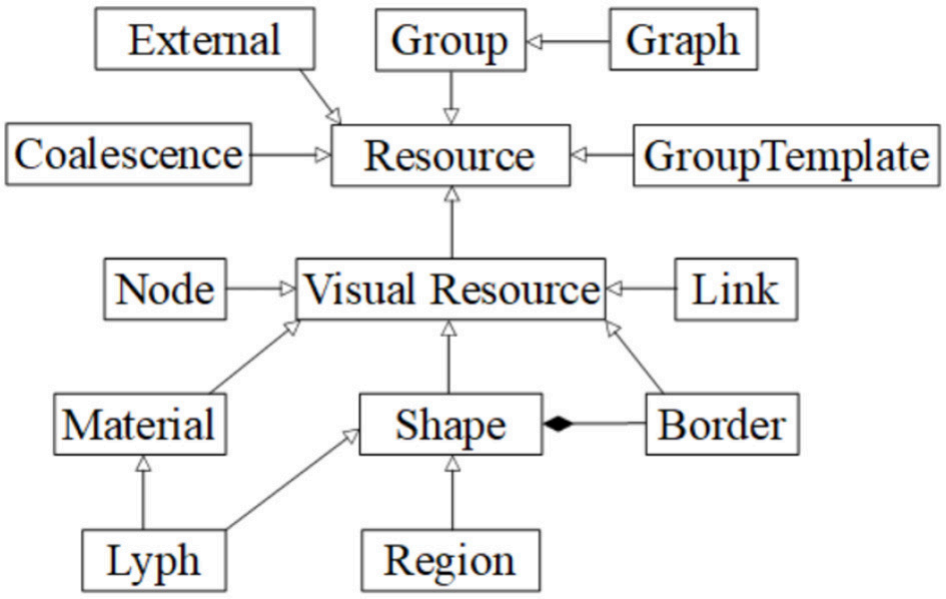
ApiNATOMY data model overview.

The ApiNATOMY KR class hierarchy (mapped out in Figure 7) defines concepts necessary to build the physiology process graph. The basic elements of such a graph are nodes (vertices) and links (edges), modeled by *Node* and *Link* classes. Other two key concepts, *Lyph* and *Material*, define composition of physiology compartments. The *Coalescense* class models coalescing assemblies. The *Region* class helps to outline model context. The notion of *Group* is used to split the model into logically-meaningful parts while *Graph* handles the representation of the model as a whole.

*External* resources are employed to annotate ApiNATOMY objects with relevant terms and definitions from existing ontologies, clinical and research databases. Abstract classes such as *Resource, VisualResource, Shape*, and *GroupTemplate* help us to generalize common properties. The latter class serves as a superclass for specifying properties of various generated groups (subgraphs) to model arborizations (*Tree* class), process edge paths (*Chain* class), embedded processes (*Channel* class) and specialized formations (e.g., *Villus* class). Finally, auxiliary classes encapsulate recurring properties of involved concepts, i.e., the *Border* class is responsible for handling content on the borders of lyphs and regions. Properties of these classes reflect the meaning of the associated physiological elements (connections, processes or material composition) and provide constraints and visual parameters that help us to assemble them into structurally correct models of physiology.

Since ApiNATOMY is as an experimental framework with evolving requirements, the ApiNATOMY toolset should be able to accommodate eventual changes in the conceptual model. For example, we can expect that a more specialized resource type is introduced to define a recurring element in some field of study or a new relationship among existing concepts is added to enable a certain type of process analysis. Hence, we looked for a declarative notation to describe and document (various versions) of a conceptual model which would also be suitable for code generation to minimize the manual effort of adapting the implementation to the updated requirements.

We chose JSON Schema specification (IETF Working Group) to define the ApiNATOMY conceptual model and further extended it to provide necessary information for code generation. The schema standard allows us to define abstract data types which are useful for auto-generated documentation, quality assurance of the client data, and automated testing. We also rely on the JSON schema to automatically produce a hierarchy of JavaScript classes for in-memory manipulation of physiology graphs. Each class manages the properties defined by the schema.

We created a minor JSON schema extension to link fields of related resources in bi-directional relationships. For example, given a sample class hierarchy in Listing 1 and an organ system model shown in Listing 2, we can recognize related properties and auto-complete relevant object definitions with missing fields as in Listing 3:

- Property *composes* of the class *Tissue* is linked via the *relatedTo* field of the extended schema to the property *composedOf* of the class *Organ*.
- Given an organ entry and, more specifically, its *composedOf* field, we can link the tissues it references, i.e., Retina, to the organ which it makes up, i.e., Eye, via its field *composes*.
- Similarly, Iris becomes *partOf* Eye since *parts* and *partOf* fields are defined as related in the class *Organ*.

**Listing 1:**
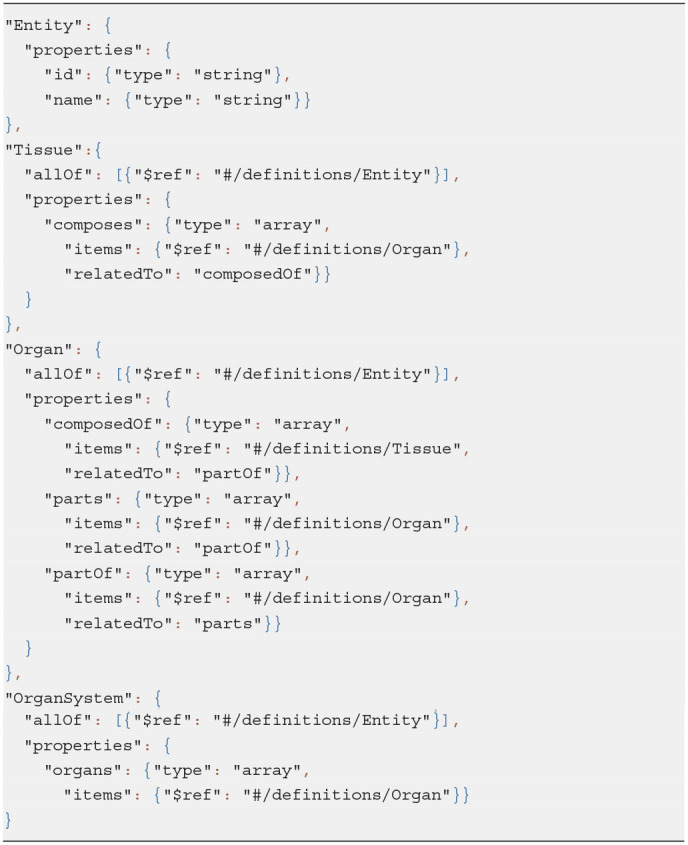
Class definitions in extended JSON schema

**Listing 2:**
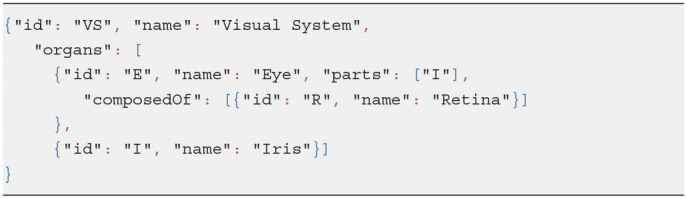
Input data: an organ system

**Listing 3:**
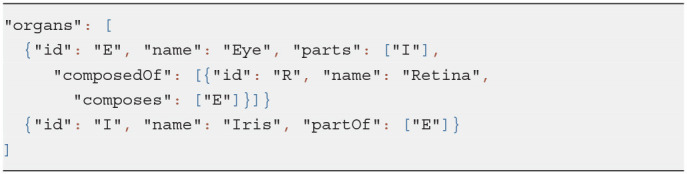
Enhanced organ definitions automatically derived from the partial input data

Thus, a user is free to choose how to insert linked data without the need to duplicate it.

### 3.1. Resources, Visual Resources, and Externals

The ApiNATOMY modeling classes have a common ancestor, an abstract class *Resource*, which defines properties present in all objects of the model. Other classes inherit from the *Resource* signature properties *id, name*, and *class*.

Resources in ApiNATOMY are defined by semantic properties which establish their physiological meaning and visualization properties which instruct the viewer tool about the desired look and layout of the model graph. To simplify visualization parameter setting, we provide means to assign values to subsets of entities in the model. Each object may contain a property *assign* with two fields: *path*, which contains a JSONPath (Gössner) expression, and *value* object, which contains a JSON object to merge with resources in the set defined by the query in the path. For example, the code in Listing 4 assigns *color* to all lyphs in a group.

The model also allows interpolation over a range of values for certain properties, in particular, users can apply color interpolating schemes and gradual distance offsets. This is done with the help of the resource's property *interpolate*. If the JSONPath query returns a one dimensional array, the schema is applied to its elements. If the query produces a higher-dimensional array, the schema is applied to all one-dimensional splices of the array. The fragment in Listing 4 colors three layers of every lyph in a group using the shades of blue, starting from the opposite side of the color array with 25% offset (to exclude very light or very dark shades).

**Listing 4:**
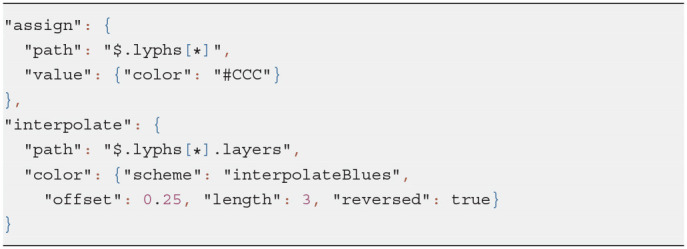
Example of assign and interpolate statements

**Figure 8:**
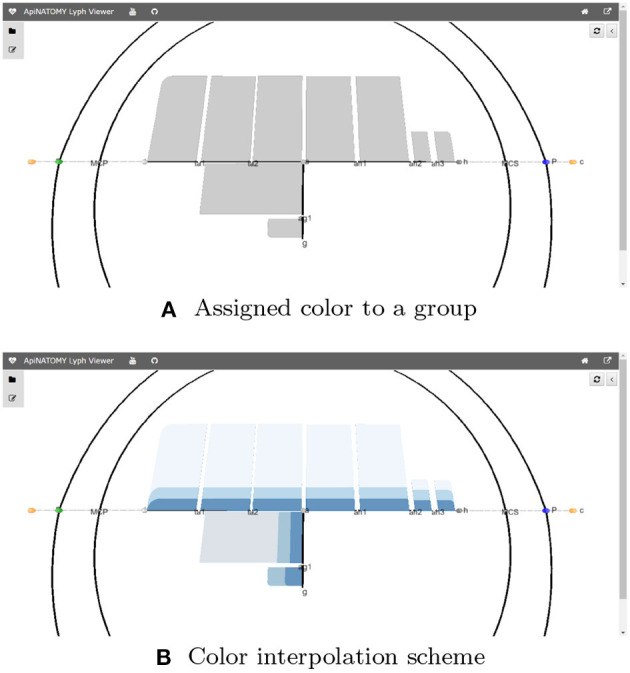
JSONPath-based assignment of resource properties. **(A)** Assigned color to a group. **(B)** Color interpolation scheme.

Figure 8 visualizes the effects of the aforementioned statements applied to the CNS lyphs.

The property *external* of each resource can be used to annotate this resource with external data. The *External* class provides fields *uri* and *type* to keep universal resource identifiers and classify the references, respectively. Among the common data sources we use in ApiNATOMY models at different scales are the Foundational Model of Anatomy (FMA) (Rosse and Mejino, 2008), Uber-anatomy ontology (UBERON) (Mungall et al., 2012), Gene Ontology (GO) (Gaudet et al., 2017), Cell Ontology (CL) (Diehl et al., 2016), Chemical Entities of Biological Interest (ChEBI) ontology (de Matos et al., 2010), and PubMed database of medical publications (National Center for Biotechnology Information).

The *VisualResource* class enlists properties common for all visual resources. Among the basic properties of this class is *color*. Relationship fields *clones* and *cloneOf* may link resources which represent the same semantic concept but must appear in two or more places of the process graph.

### 3.2. Materials and Lyphs

*Material* and *Lyph* are two key concepts in the ApiNATOMY framework. *Material* is more suitable for modeling chemical elements or substances as an aggregate whole while *Lyph* defines topological organization (i.e., layers of tissue material constituting a body conduit vessel) or elements with an emphasis on a shape. The same substance sometimes can be modeled both as a material and as a lyph. Listing 5 models *blood* both as regional and constituent parts of the “Right Ventricle” heart chamber model.

**Listing 5:**
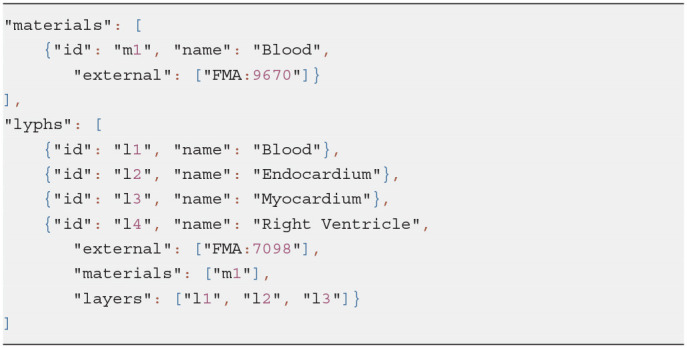
Defining materials and lyphs

Technically, lyph is subtype of material with spatial constraints. Both materials and lyphs can contain other *materials* or be included into hosting material via a related property *materialIn*. A material can also be transported by a channel (special type of a process) when its property *transportedBy* is set up.

### 3.3. Shapes, Borders, Lyphs, and Regions

A shape is an abstract concept that defines properties and relationships shared by the ApINATOMY resources for modeling physiology compartments, namely, lyphs, and regions. The property *internalNodes* accumulates nodes positioned on a shape. Such nodes are projected to the shape's surface and positioned to stay within boundaries of the shape (e.g., by attracting to its center). The property *internalLyphs* is used to define the inner content of a shape, i.e., neurons within the neural system parts. The related property, *internalIn*, indicates a shape (lyph or region) the given node or lyph belongs to. The *hostedLyphs* property is similar to the *internalLyphs* but uses a different layout method: hosted lyphs get projected on the container lyph plane and forced to stay within its boundaries.

**Figure 9:**
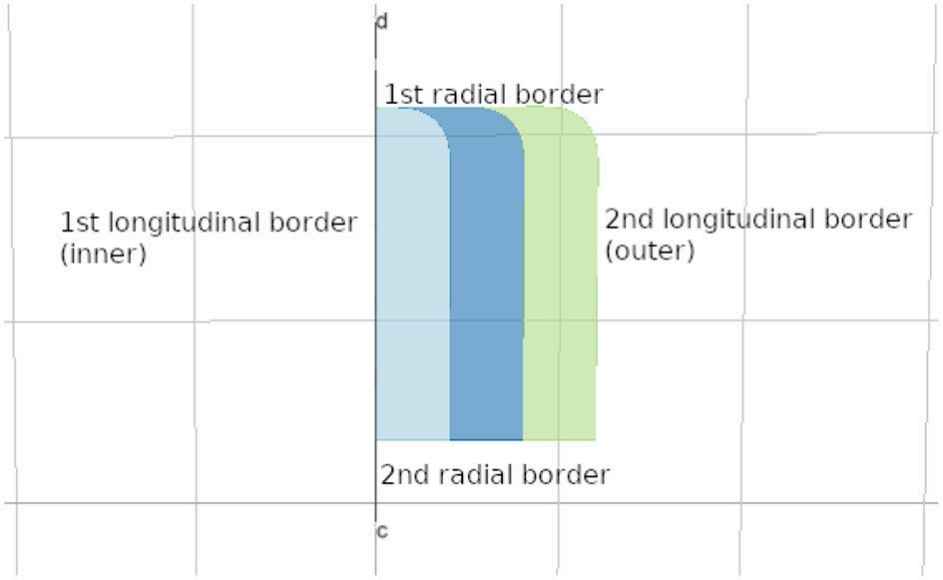
Lyph's border.

An important part of the shape abstraction is its *border* (see Figure 9). The shape's border is a resource with its own identifier, name, and external annotations. It refers to the hosting shape via its property *host* while the shape can access it via its *border* field. We auto-generate borders for lyphs and regions in the model and merge inline objects defining border content within the hosting shape with the generated resources.

The lyph's border is closely related to its *topology*. The topology value TUBE represents a conduit with two open ends. The values of BAG- and BAG+ represent a conduit with one closed end, where − and + represent the polarity of the closed border. Finally, the topology value CYST represents a conduit with both closed ends.

**Figure 10:**
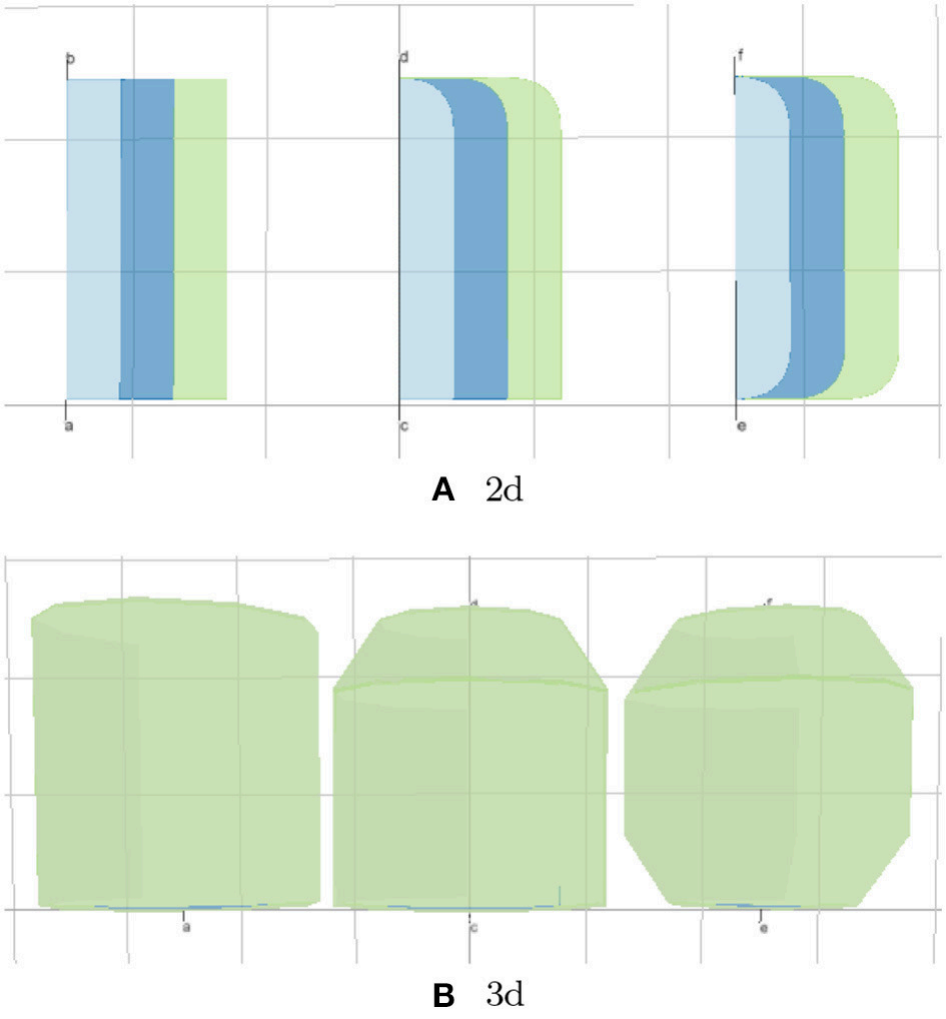
**(A, B)** Lyph topology types: TUBE, BAG, and CYST.

In the model viewer, lyphs are depicted as 2d rectangles either with straight or rounded corners, where the straight corners correspond to open-ended radial borders and rounded corners represent the close-ended radial borders (see Figure 10A). The lyph's axis of rotation is normally aligned with the edge conveyed by a lyph or, in the case of layer lyphs, with its hosting lyph. All borders can be accessed via the lyph border property *borders* which expects an array of four objects. Listing 6 shows an excerpt from the BG model (see section 5 for more details) which defines a lyph with content (node) on its second radial border. If a lyph border conveys processes or contains nodes as in this case, we associate a *Link* resource with the lyph's border segment.

**Listing 6:**
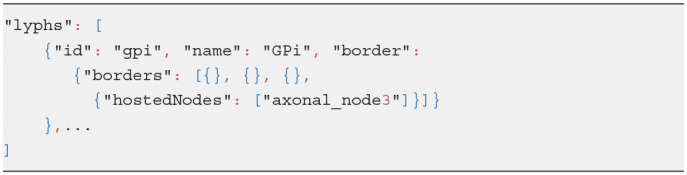
Lyph's border content

The topological organization of a lyph is defined by its *layers*. A *layer* is a lyph that represents a tissue within another tissue and rotates around the axis of its hosting lyph, to which it refers via its *layerIn* field. By default, all layers get the equal display area within the main lyph. The percentage of the display area a layer occupies can be regulated via its *layerWidth* parameter.

Often a model requires many lyphs with the same layer structure. To simplify the definition of such lyphs, we introduced a notion of the lyph *template*. A lyph with a Boolean property *isTemplate* set to true serves as a prototype for all lyphs defined as its *subtypes*. Equivalently, lyphs inherit their layers and other transferable properties from their *supertype*. The hierarchy of lyph derivation via subtyping or supertyping relationships can be traced with the help of the relationship graph introduced in section 4.

The center of the lyph's 1st longitudinal border is placed to the center of the link it *conveys*. When lyph is a layer in another lyph, its axis of rotation depends on the hosting lyph. Some additional properties exist to set the angle of rotation of a 2d lyph around its axis.

The lyph's dimensions depend on the length of the conveyed link and can be controlled via the *scale* parameter. The ultimate visual dimensions of a lyph object are provided by its *width* and *height* properties while *thickness* and *length* refer to the anatomical dimensions of the physiological resource it models (a range of values in negative powers of 10, e.g., [10^−6^..10^−4^]).

The Boolean property *create3d* indicates whether the editor should generate a 3d object for the given lyph. The 3d view gives the most accurate representation of a lyph but makes it difficult to analyze its inner content. Figure 10 shows 2d and 3d visualizations of lyph topological types.

Lyphs also contain properties linking them to composite assemblies, e.g., *inCoalescences* refers to coalescence assemblies that include a given lyph, *channels* lists channel assemblies housed by the current lyph, *bundles*, and *bundlesTrees* establishes relationships with links and trees passing through the lyph.

*Regions* are flat auxiliary shapes which provide context to the model. Region internal content is similar to the content of a lyph. Regions are static and their positions are given in 2d coordinates. The border of the region can include any number of segments (links), unlike lyphs which always have four sides. Examples of models with regions are available in our project's test directory (Kokash).

### 3.4. Nodes and Links

In the ApiNATOMY graph, nodes (vertices) connect links (edges) which are conveyed by lyphs (edge labels). Each node refers to the links it joins via its fields *sourceOf* and *targetOf*.

The positions of nodes in the ApiNATOMY models in most cases cannot be explicitly set by model authors due to the absence of such data. Hence, to produce an intuitively clear uncluttered graph visualization, we rely on our custom model rendering algorithm. The algorithm is based on the force-directed layout in which some node positions are constrained by entity relationships.

Node definition provides parameters to control its appearance and/or position in the force-directed environment. The radius of the sphere representing the node in the model visualization is computed based on the value of the node's *val* property. A number of properties such as *charge* and *collide* allow users to tune the forces applied to a specific node. Node positions can be controlled via the *layout* property. The value of each coordinate is expected to be in the range [−100..100] and represents the percentage of the length from the center of coordinates to the end of the plot along the respective axis. The actual coordinates are then computed depending on a selected scaling factor. Coordinates of nodes marked fixed are rigidly set to match the *layout* values. For other types of nodes, the layout only defines the position the node is attracted to while its actual position *(x, y, z)* may be influenced by other factors, i.e., global forces in the graph, link rigidity, positions of other nodes, and so on.

It is important to retain the containment and spacial adjacency relationships among resources. Several properties are used to position nodes on a link, within a lyph or on its surface. It is also possible to define the position of a node based on the positions of other nodes. For example, to place a node on a link, we associate the link with the node via the node's property *hostedBy*. The property *offset* can be used to indicate the offset in percentage from the start of the link. For example, the node definition in Listing 7 instructs the tool to position the node nLR at the quarter of the length of the link LR.

**Listing 7:**
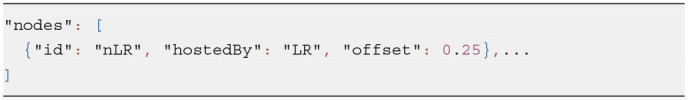
Node on a link

An alternative way to get the same result is to include the node to the *hostedNodes* property of the link.

Links connect graph nodes and perform a number of functions in the ApiNATOMY framework, most notably, they model process flow and serve as rotational axes to position and scale conveying lyphs. By default, all links are drawn as straight lines, this corresponds to the *geometry* property with value link. To apply another visualization method, a user should explicitly set the link's geometry to a desired value. For example semicircle produces a spline that resembles a semicircle, rectangle draws splines resembling a semi-square connector with rounded corners, spline produces link connectors which smoothly join preceding and following links, and path draws graph edges bundled together by the edge bundling algorithm (Holten and van Wijk, 2009). Auxiliary invisible links are not displayed in the process graph but serve as axes for the lyphs they convey. In certain cases, we automatically create invisible links (e.g., when an internal lyph is not conveyed by any existing link, when lyph's border segment hosts nodes). A link, regardless of its geometry, can be drawn using dashed or thick lines.

The property *length* defines the desired distance between the link's ends as percentage of the maximal allowed length. The force pushes the link's source and target nodes together or apart according to the desired distance. *Collapsible* links are links which appear only if their ends are constrained by the visible entities, e.g., to connect node on lyph borders. If this is not the case, the source and target nodes of the collapsible link are attracted to each other until they collide to look like a single node.

### 3.5. Coalescence

**Figure 11:**
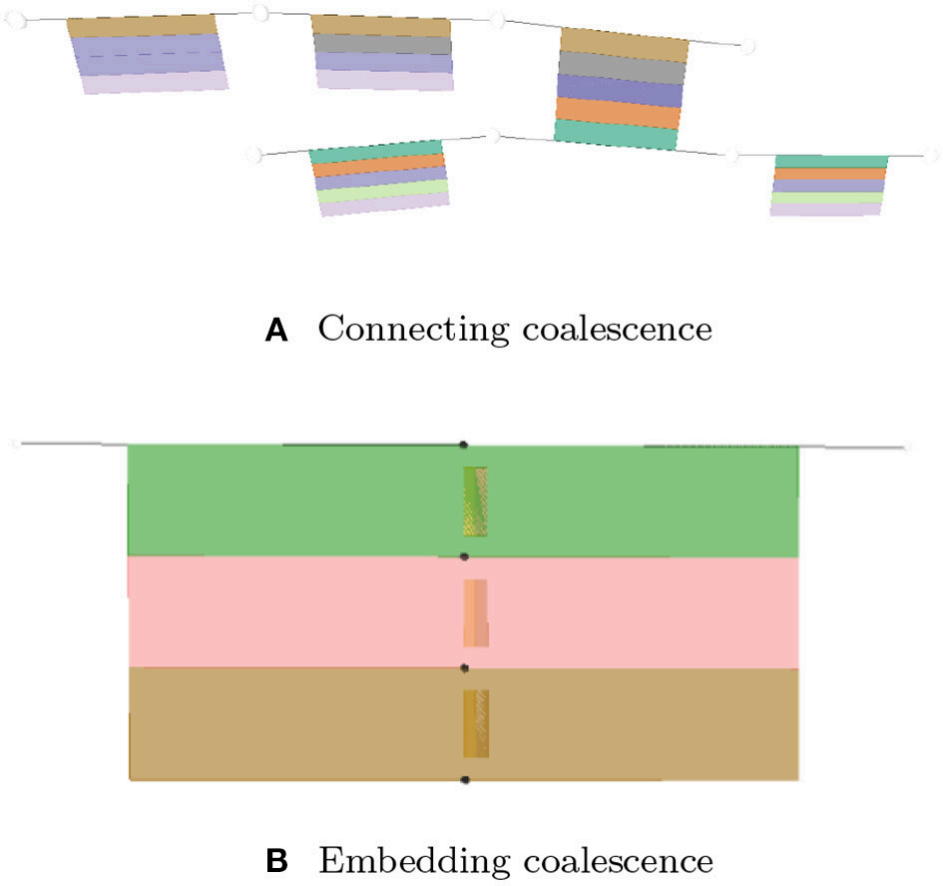
Visualization of coalescing lyphs. **(A)** Connecting coalescence. **(B)** Embedding coalescence.

A *coalescence* creates a fusion situation where two or more lyphs are treated as being a single entity. There are two types of coalescence, both describing material fusion. In both cases, coalescence can only occur between lyphs that have exactly the same material composition:

- *Connecting* coalescence represents a “sideways” connection between two or more lyphs such that their outermost layer is shared between them (as shown in Figures 4, 5, 11A).
- *Embedding* coalescence (Figure 11B) represents placing of a contained lyph into a container lyph such that the outer layer of the contained lyph merges into the container lyph as a means to globally associate measurements, that are local to the contained lyph, with the container lyph.

The *Coalescence* class introduces fields *topology* and *lyphs*. These fields are used to specify the type of the coalescence, CONNECTING or EMBEDDING, and the coalescing lyphs, respectively. In connecting coalescence, outermost layers of coalescing lyphs appear to share the same visual object. In embedded coalescence, the outermost layers of coalescing lyphs blend with the corresponding layers of the housing lyph.

Although semantically the lyph order in the coalescence definition is not important, in the viewer, the first lyph is treated in a special way. While connecting the graphical depictions of coalescing lyphs, all subsequent lyphs are pushed by the force-directed layout to approach the master lyph and realign their outermost layers to match the outermost layer of the first lyph. In the case of the embedded coalescence, the first lyph is the *housing* lyph—it contains the other lyphs in the coalescence.

If a coalescence resource is defined on abstract lyphs (lyph templates), it is considered abstract and works as a template to generate coalescences among lyphs that inherit the abstract lyphs.

### 3.6. Groups and Graphs

The *Group* class defines a subset of entities in an ApiNATOMY model which have a common semantic meaning and/or a distinct set of visual characteristics. A group includes lists of resources of any type via the dedicated fields: *nodes* for resources of type *Node, links* for resources of type *Link*, and so on. Groups can include nested subgroups and refer to the resources defined anywhere in the model. For example, Listing 8 shows a group of blood vessels that consists of two subgroups: (a) arterial and (b) venous.

**Listing 8:**
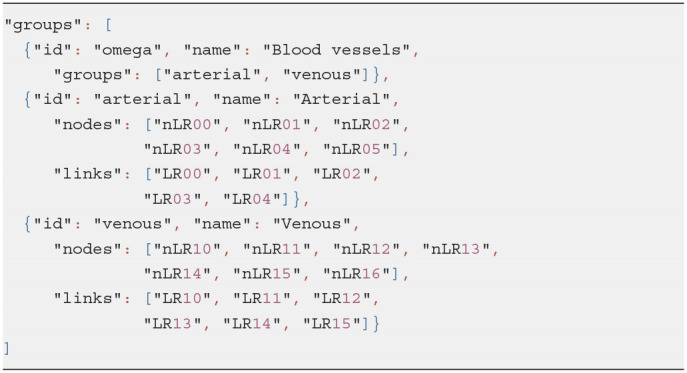
Group composition

The model viewer allows users to inspect each group in isolation or analyze the interaction among selected combinations of resources without overloading the view with unnecessary information.

The *Graph* class describes the top-level group that, in addition to all the group fields, contains *config* field and model meta-data such as *created* and *lastUpdated* dates, *author* and *version*. The *config* is not part of the semantic physiology model but it can be used to define user preferences regarding the visualization of a given model, e.g., which groups to display by default, whether to use resource identifiers or names as labels in the graph, etc.

### 3.7. Group Templates

A *group template* represents an abstract class to model group generation patterns. The generated group is accessible from the template via its property *group*. Currently, ApiNATOMY supports five types of group templates requested by the modeling experts. In this section, we describe two of them: *Tree* and *Channel*.

The *Tree* group template allows users to specify a root of a tree via an optional property *root*. If the root node is not specified, it is either automatically generated or assigned to coincide with the source node of a link that represents the first level of the tree. We define the *level* of a tree branch as the number of edges between the target node of the link and the root. A user may specify the required number of levels in a tree via its property *numLevels*. Given the desired number of levels, the model viewer can generate a *group* resource to represent the tree. If the property *lyphTemplate* is set up, the generated links for each tree level conveying lyphs which are sub-types of the lyph template.

Alternatively, the tree branches can be specified, partially or fully, via the property *levels* which expects an array of (partial) link definitions corresponding to the tree levels. If this array contains an empty object, the missing tree level branch is auto-generated. If instead it points to an existing link, the corresponding link is joint to become a branch in the tree. If the source, target, or both ends of the link resource are not specified, they are auto-generated. Otherwise, it is expected that incident links (connected tree branches) share a common node; the tool will issue a warning if this is not the case.

Listing 9 demonstrates how to define trees using both *lyphTemplate* and *levels* properties. A combination of both approaches may help modelers to reduce an effort while specifying multi-level trees where the majority of the branches consist of the same lyphs (i.e., involve the same tissue structures) while others are characterized by unique features that need to be modeled individually.

**Listing 9:**
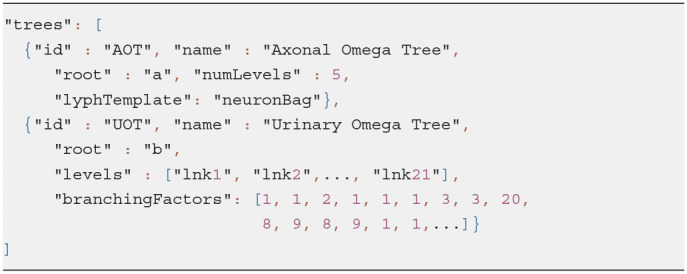
Tree template examples

The generated sub-graphs for tree templates defined above would look like liner chains of enumerated links. We refer to such trees as *canonical*, meaning that they define the basic structure necessary to generate a branching formation. The branching can happen at each level of the canonical tree, and the number of branches per level can be specified in the *branchingFactors* array. Furthermore, the property *numInstances* contains a number of branching tree instances produced from a canonical tree specification. The generated tree instances are available via the read-only property *instances* in the generated model.

**Figure 12:**
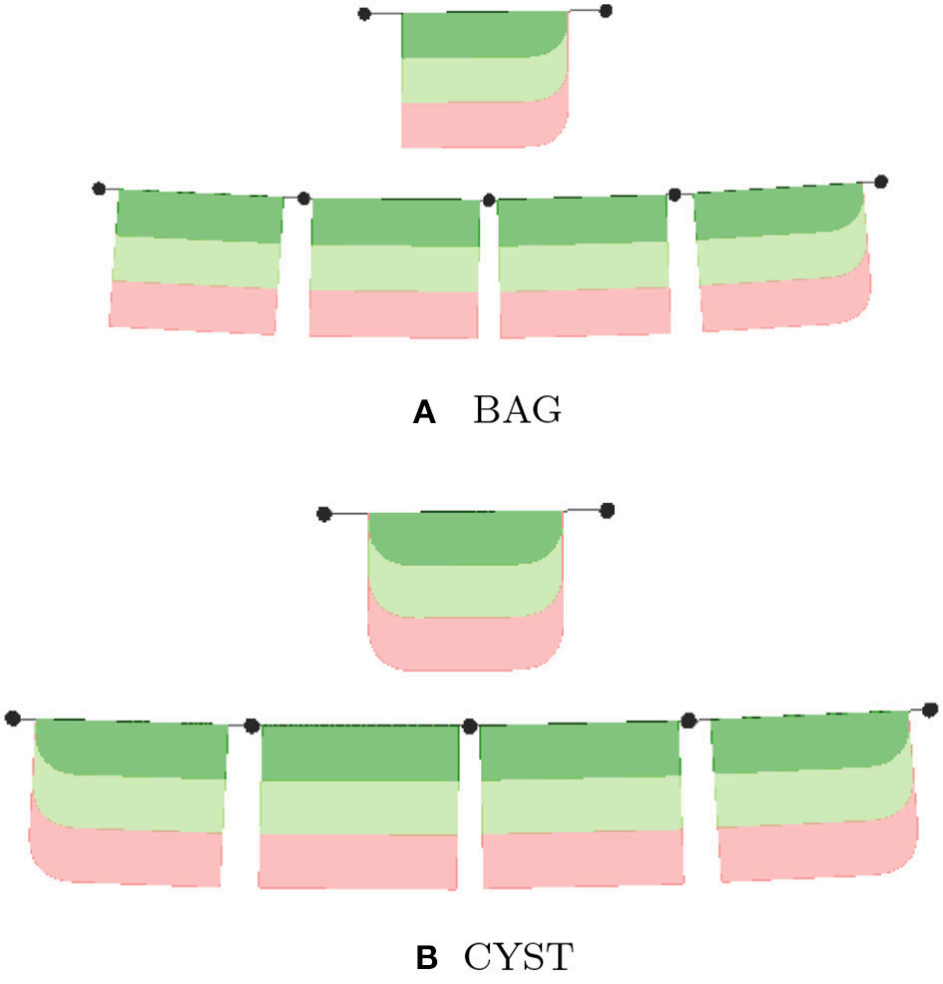
Deriving topology of tree level conveying lyphs from lyph template. **(A)** BAG, **(B)** CYST.

Generally, lyphs originating from a lyph template inherit its topology. However, the lyphs conveying the tree edges work as a single conduit with topological borders at the start and the end levels (root and leaves) of the tree compliant with the borders of the lyph template. The topology of lyphs on tree edges is defined as shown in Table 1. Figure 12 illustrates the difference in generated trees from lyph templates with the same structure but different topology: (a) template of type BAG+ results into a tree with the BAG+ lyph at the last level. (b) template of type CYST translates into a tree with the lyphs of type BAG- and BAG+ on the first and last tree levels.

**Table 1:** Omega tree lyph topology.

**Lyph template**	**Radial borders**	**Tree**	**Level 1**	**Levels 2.N-1**	**Level N**
*TUBE*	Both open	*TUBE*	*TUBE*	*TUBE*	*TUBE*
*BAG* ^−^	1st closed	*BAG* ^−^	*BAG* ^−^	*TUBE*	*TUBE*
*BAG* ^+^	2nd closed	*BAG* ^+^	*TUBE*	*TUBE*	*BAG* ^+^
*CYST*	Both closed	*CYST*	*BAG* ^−^	*TUBE*	*BAG* ^+^

The *Channel* group template provides fields to define a complex specialized assembly to model membrane channels incorporated into given housing lyphs. To create membrane channel components, a model author has to provide:

- an identifier for a channel (and, optionally, other basic resource properties);
- identifiers of housing lyphs. A *housing lyph* is a lyph of at least three layers representing a cell or an organelle such that the middle layer of this lyph is a membrane (e.g., Sarcoplasmic Reticulum);
- the material payload conveyed by the diffusive edge.

Listing 10 shows an example of the channel template definition: a membrane channel SERCA exchanger housed by lyphs derived from the lyph template Leukocyte (see Figure 4).

**Listing 10:**
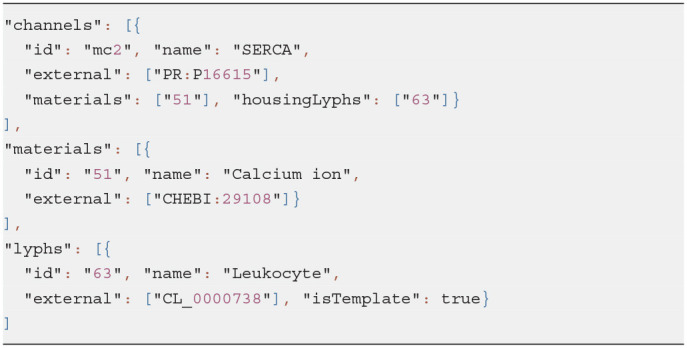
Example of a channel template

The identifiers of the housing lyphs and materials conveyed by the diffusive edges are specified in the resource properties *housingLyphs* and *materials*, respectively. A lyph refers to the channel it houses via its property *channels*.

Given these data, the model generator creates three tube lyphs representing the three segments of the membrane channel (MC): *internal, membranous* and *external*. Each of the MC segments consists of three layers: *innermost* (content), *middle* (wall), and *outermost* (the same material as the lyph that contains it).

**Figure 13:**
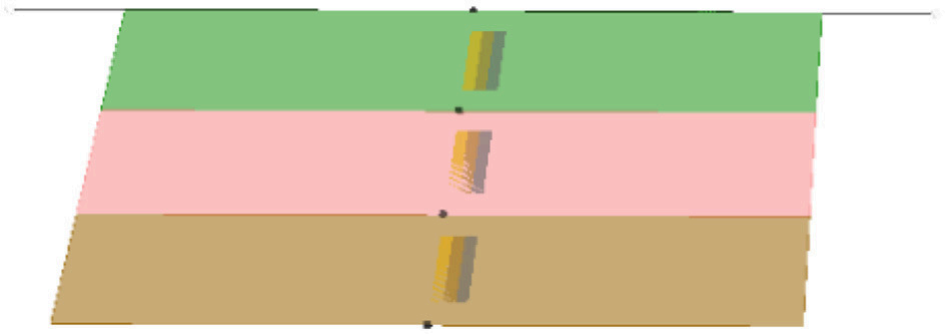
Housed membrane channel.

The assembly generated given such a template consists of at least 20 resources: 4 nodes, 3 links, 3 lyphs with 3 layers each, 3 embedding coalescences, and a group encompassing all the above (see Figure 13). The three MC segments are housed respectively in the three layers of the housing lyph. We automatically create constraints to position channel nodes on the borders of the layers of the housing lyph, so in practice several auxiliary border resources are created. If the housing lyph is a template, these resources are replicated for each channel instance housed by a lyph instance originating from the lyph template.

The third layer of each MC segment undergoes an embedding coalescence with the layer of the housing lyph. Each of the three MC segments conveys a diffusive edge such that both nodes of the edge conveyed by the MC segment in the second (membranous) layer are shared by the other two diffusive edges. Diffusive edges are associated with the links conveying the membrane channels (the link's *conveyingType* is set to DIFFUSIVE) and the material in the channel object is copied to the *conveyingMaterials* property of the link.

## 4. Model Generation

ApiNATOMY users describe key elements of a physiological model, their relationships, and layout constraints in an input file. These models may be incomplete or incorrect (i.e., contain typos, undefined references, unexpected values, etc.). We use the model schema and custom validation rules to discover potential problems in the input model prior to its visualization. If no critical errors are found, the model viewer proceeds with expanding templates, generating missing resources, and drawing the model graph. Here we describe the key stages of model generation in order to prepare it for visualization. A number of resource relationships are established during this process to enable accessibility of model objects.

**Figure 14:**
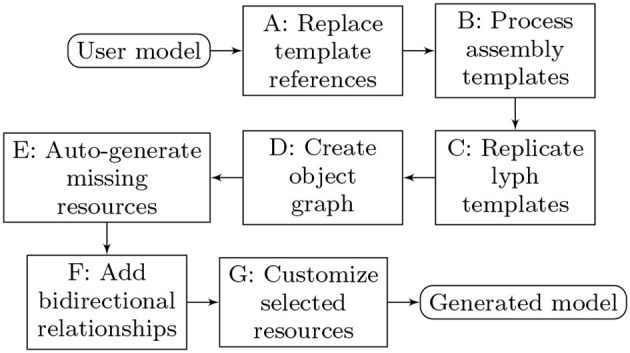
Model generation pipeline.

**Figure 15:**
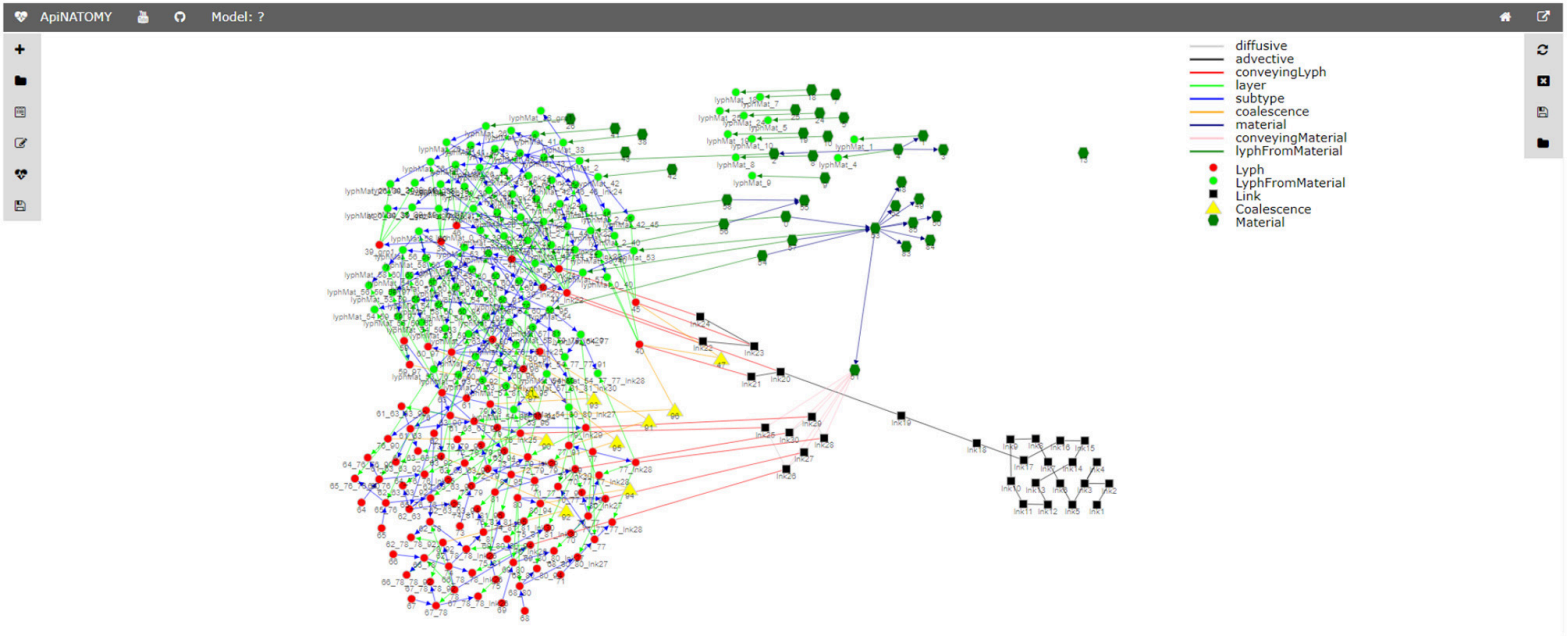
Relationship graph.

The overall model processing pipeline is presented in Figure 14. The list below explains model transformation actions at each step:

**A: Replace template references**. Many resources that domain experts need for modeling are abstract assemblies of physiology subsystems (materials, cells, neuron pathways). It is convenient to specify such resources once and place them to the context they are used in as many times as needed. On the other hand, the ApiNATOMY model viewer creates a visual artifact for each unique visual resource (lyph, node, link) in the model. Hence, we have to replace the abstract templates with resource instances that inherit the majority of their characteristics from the templates. The tool identifies references to materials and lyph templates in all the fields that are expected to contain lyphs as building parts, automatically creates lyph instances with necessary characteristics and replaces abstract references with instance references. All derived or cloned resources within this step can be overviewed with the help of the relationship graph (see Figure 15).
**B: Process assembly templates**. If composite assembly templates such as trees or channels are present in the model, we generate corresponding graph structures and include all created resources (nodes, links, lyphs, etc.) to the main graph (or a parent group containing nested models). The procedure replicates lyph templates to all generated edges (links) and assigns their topology to define overall boundaries of the conduits. At the end of this stage, the lyph template is linked to the newly created blank lyphs.
**C: Replicate lyph templates**. At the next step, we process lyph templates: all subtypes of a lyph template inherit its layers, color, size-related properties, namely, scale, height, width, length, and thickness (unless they are overridden for the lyphs individually), external annotations, constituent materials, etc. Visual resources such as layers of lyph instances originating from lyph template layers are linked together via the *cloneOf* property.
**D: Create object graph**. After auto-creating resources originating from group and lyph templates, the model's graph structure is almost ready to be visualized. Hence, we create ApiNATOMY model objects and replace string identifiers with the corresponding object references. Lyph and region borders are auto-created and merged with user-defined border content. Missing links and nodes for internal lyphs are auto-created. Finally, we perform group inclusion analysis and include nested group resources into parent groups.
**E: Auto-generate missing resources**. As the result of the previous step, we obtain a complete map of all model resources regardless of where they were defined, i.e., all references can be resolved. If the tool detected IDs without corresponding resource definitions, such objects are auto-generated. A user can detect which resources were created by checking their property *generated* (it will be set to true) for such objects) and/or inspecting the logs.
**F: Add bidirectional relationships**. In a bidirectional relationship, each entity has a relationship field or property that refers to the other entity. To be able to fully connect related resources, we rely on the model schema to supplement resource definitions with missing fields.
**G: Customize selected resources**. At the final stage, we perform model customization via the JSONPath queries in *assign* and *interpolate* properties. This is done in two steps. First, we create dynamic relationships by replacing IDs in the fields with object references (only IDs of known objects should be used in the dynamic *assign* expressions, unresolved IDs will be ignored). Second, we complete model customization by assigning qualitative properties to resources selected by JSONPath queries for every resource in the model with the *assign* or *interpolate* properties.

A number of critical and non-critical errors can be detected during the model generation process. The viewer has been designed to be fault-tolerant, so it attempts to display the (correct part of the) model and exposes the log messages with all detected errors and warnings for user inspection. The generated model and the resource map (a list of all resources in the model, both user-defined and auto-generated) in the JSON format can be exported and integrated with external knowledge bases.

The implementation of the model generation steps listed above is completely abstracted from the semantic meaning of the resource classes. The implication of this is that the ApiNATOMY model viewer can easily evolve—one can define new types of resources or introduce new relationships simply by adding the corresponding definition to the model schema, and, if needed, write code to draw a visual object representing the new concept.

The relationship graph (see Figure 15) is an auxiliary structure that allows users to trace the resource derivation process described above and inspect the relationships among the key resources in the model. For example, this structure can be used to find lyphs that sub-type a given abstract lyph or include a given material. All resources in the relationship graph are represented by graph nodes (visualized using colored shapes according to the type of the resource) while their relationships correspond to the graph links (visualized using colored lines where each color stands for a certain type of the relationship). The view will further evolve to become an interactive instrument for model validation, with the ability to explore various relationships among selected resources.

## 5. Display Layout

The Basal Ganglia (BG) resource graph (illustrated in Figure 5) includes four fields: nodes, links, lyphs, and trees (see Listing 11). The context lyphs, Putamen, GPe, and GPi, are modeled as internal lyphs of the BG lyph. This implies that they are positioned on a grid within their container lyph, and the corresponding parameter, *internalLyphColumns* is set to indicate that the grid contains three columns. The number of rows in such a grid depends on the number of internal lyphs within the container. Other space allocation methods for internal lyphs may be employed. In particular, spatial constraint-preserving template-based treemaps (Kokash et al., 2014) appear to be particularly useful to draw internal lyphs of various size with adjacency constraints. For brevity, we skip the definition of three lyphs modeling layers of a neuron and show definition of only one BG's internal lyph, GPi. The links to convey internal lyphs as well as their source and target nodes are auto-generated.

**Listing 11:**
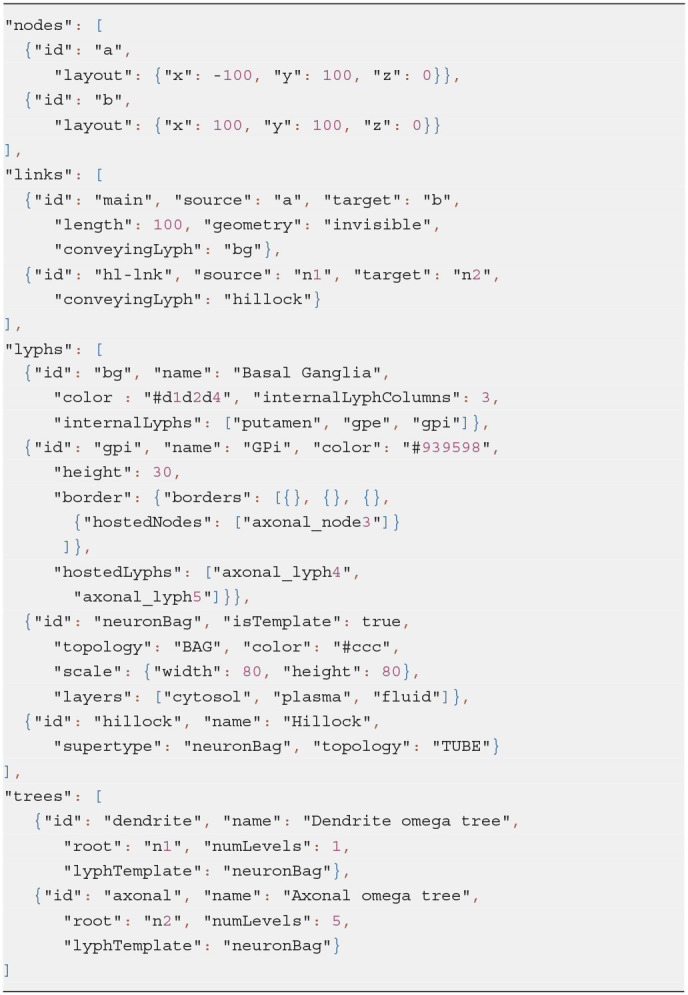
JSON specification of Basal Ganglia

The *trees* property is an array with two objects defining the dendrite 1-level tree and the axonal 5-level trees. The tree root nodes are named n1 and n2, these names are explicitly given while defining the conveying link for the axon hillock. By referencing them in the tree model, we indicate that the roots of the axonal and dendrite trees coincide with the ends of the hillock.

All auto-generated ApiNATOMY resources are assigned identifiers which can be used to integrate such resources to the rest of the model. It is useful to know that identifies for auto-generated tree parts are formed using the following patterns:

1. *$tree*.*id*_node*$index*,
2. *$tree*.*id*_lnk*$index*
3. *$tree*.*id*_lyph*$index*,

where *$tree*.*id* is the identifier of the tree, and *$index* refers to a tree level. Keeping in mind these patterns, we can position the axonal tree node (axonal_node3) on the 2nd radial border and project two axonal tree level lyphs (axonal_lyph4 and axonal_lyph5) to the surface of the GPi lyph via the border's property *hostedNodes* and the lyph's property *hostedLyphs*.

The *lyphTemplate* field refers to an abstract lyph neuronBag which defines the structure of lyphs conveyed by the trees. Due to this setting, all generated lyphs conveyed by tree edges inherit 3 layers each and occupy the square area with a side length equal to 80% of their axis length. Note that the hillock lyph also derives its structure from the neuronBag template, as indicated by its property *supertype*. The lyphs conveyed by the tree edges are seen as a single conduit with topological borders at the start and the end levels (root and leaves) compliant with the borders of the lyph template. In the BG model, the lyph template is of type BAG, hence, the conveying lyphs at the end levels are also of the type BAG. The topology of the hillock is explicitly defined as TUBE to override the inherited topology from the template.

**Figure 16:**
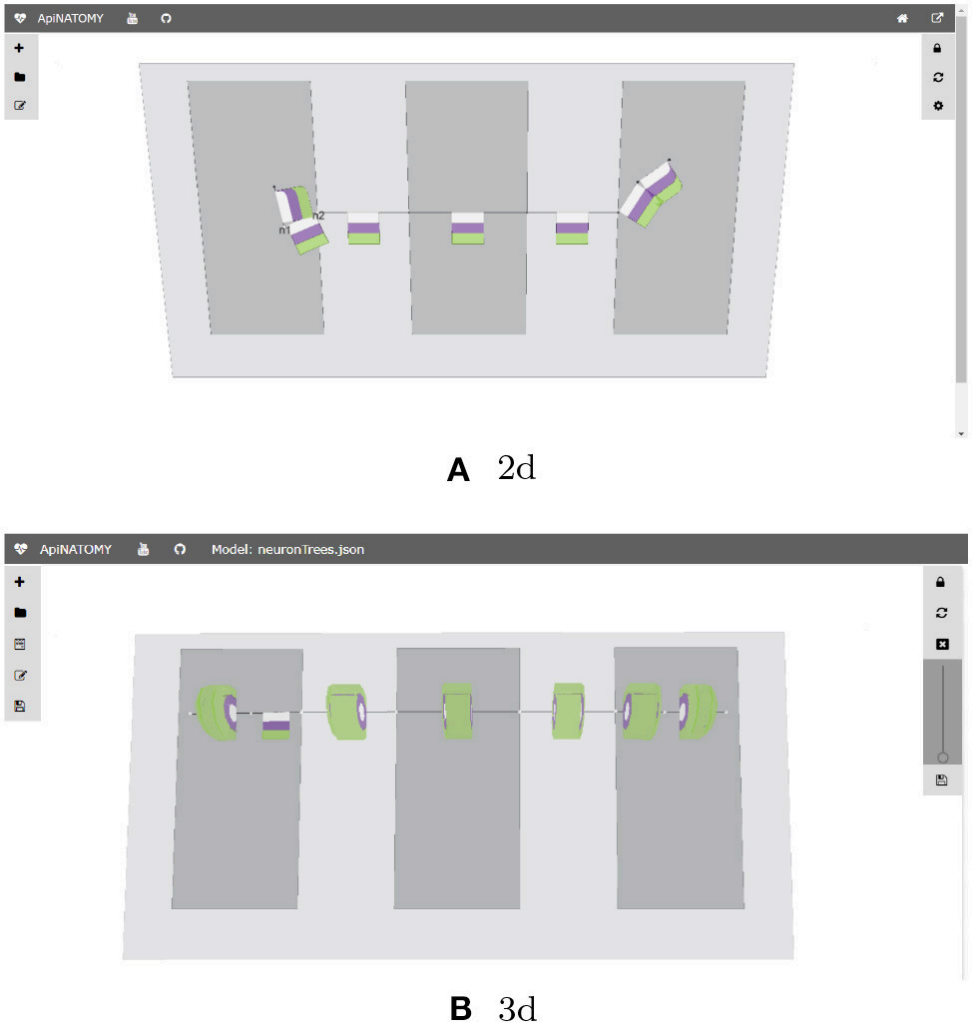
Generated ApiNATOMY visualization of BG. **(A)** 2d, **(B)** 3d.

With the conceptual model and the associated post-processing pipeline presented in this paper we were able to produce an elaborate rendering from a minimalist definition. The generated visual layouts in 2d and 3d are shown in Figure 16. The input model of the BG defined by our physiology expert consists of 15 JSON objects with 3–6 properties each. The generated model used for the visualization consists of 132 JSON objects with 10–20 fields each.

Listing 12 shows an excerpt of the Urinary Tree (UoT) model specification. For space reasons, we omit most of the objects defining visual resources. The listing emphasizes the use of nested groups to encompass the subsystems within the main graph and annotate them with suitable ontology terms: “nephron” and “loop of Henle” groups are linked to the corresponding UBERON (Mungall et al., 2012) terms. Nested groups enable complex model assemblies from existing ApiNATOMY models. They can also be toggled on and off in the model viewer to allow users focusing on a certain part of the model.

**Figure 17:**
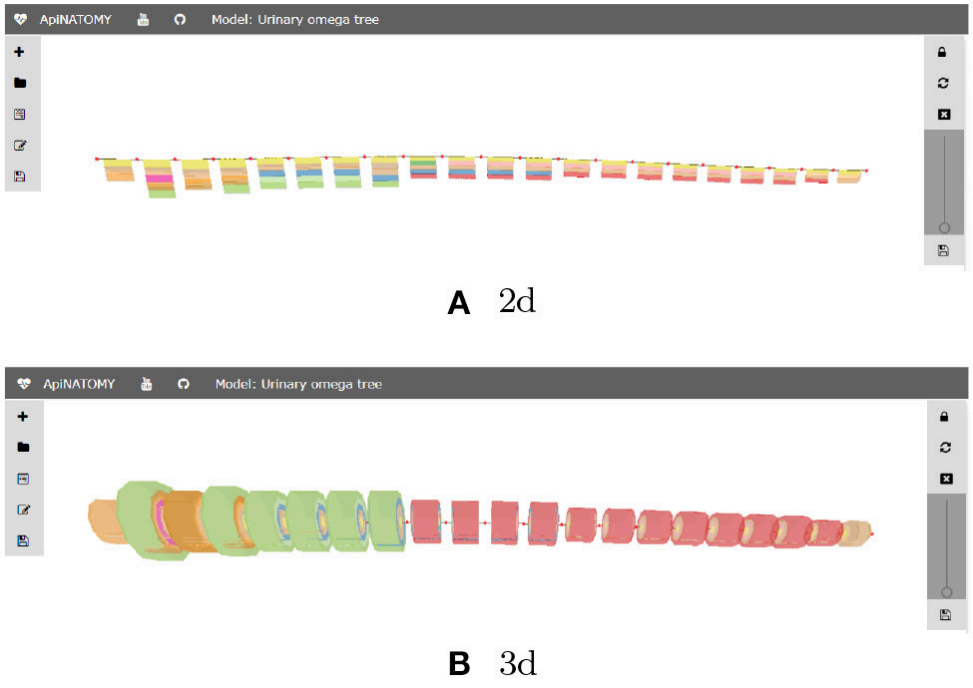
Generated ApiNATOMY visualization of UOT. **(A)** 2d, **(B)** 3d.

**Figure 18:**
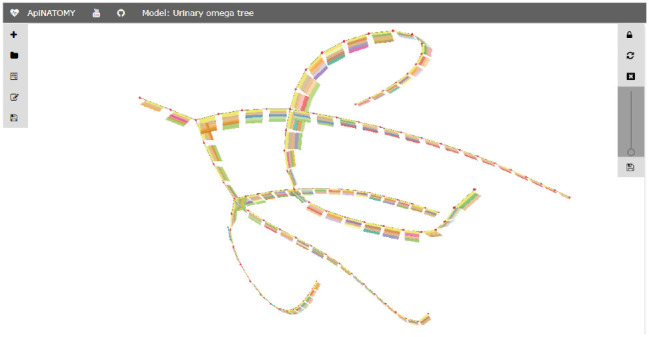
Fragment of an UOT instance.

For illustration purposes, we define the *assign* property to customize the appearance of nodes: with the help of this statement, the color of all nodes in the model is set to red. The corresponding visual renderings of the UOT canonical tree in 2d and 3d are shown in Figure 17. Figure 18, a snapshot of the UOT instance with branching in levels 3 and 7, shows a small subset of the 10^8^ visual elements of the fully branched urinary tree.

**Listing 12:**
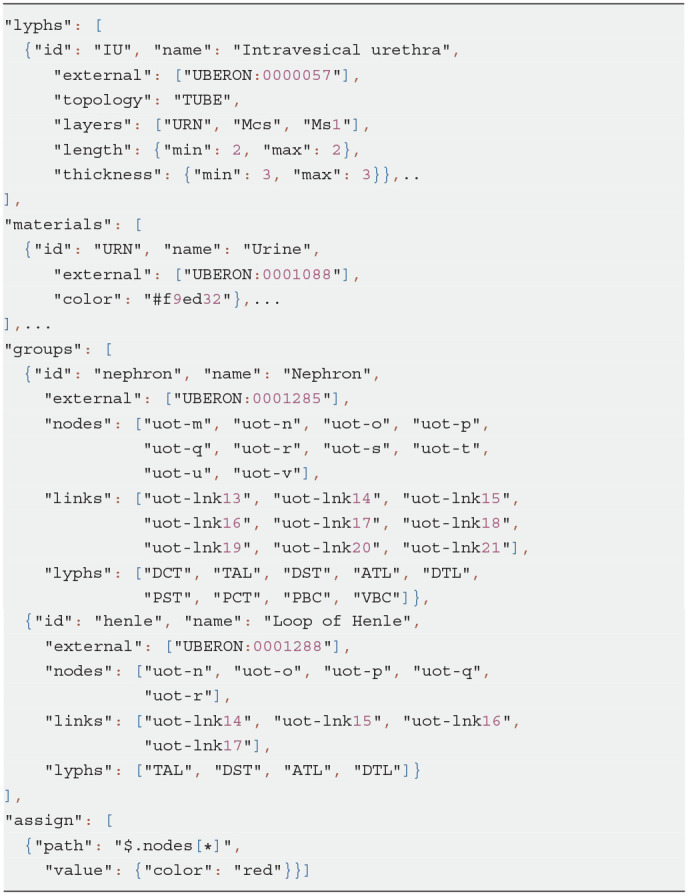
JSON specification of Urinary Omega Tree

## 6. Discussion

The ApiNATOMY methodology offers a ubiquitous approach to multi-scale route modeling by offering abstractions and structural patterns for process description in biomedicine. The purpose of the presented ApiNATOMY KR model is to improve cross-scale data integration, navigation, and mapping.

The biomedical community has recognized the need for technical integration of existing resources into multi-scale interoperability platforms. Such platforms (Eissing et al., 2011; Sarwar et al., 2019) enable modeling and analysis of physiological phenomena, information discovery, simulation of disease progression in virtual organisms and their responses to stimuli. An in-depth review of approaches on multiscale modeling of biological systems, quantitative biomedical engineering methods, and current challenges in the field can be found in Walpole et al. (2013). Various perspectives and views on physiological systems modeling, simulation, and control are also discussed in Michmizos and Nikita (2012).

The aforementioned tools mainly focus on mathematical aspects of modeling while semantic and visual aspects are considered secondary. In contrast, ApiNATOMY provides means to define elaborate structural (tissue composition, assemblies, arborizations) and interaction patterns (coalescing assemblies, adjacency, connectivity) as well as to automatically generate visual schematics which conform to the specified constraints.

On the KM side, our work is closely linked to *biological ontologies* (Lambrix et al., 2007) and *biological KM* (Antezana et al., 2009). Ontologies are widely employed in systems medicine and systems biology to define the basic terms and relations and provide the basis for interoperability between systems as well as for search, integration and exchange of biomedical data. Dedicated domain-agnostic languages for specifying ontologies such as OWL Web Ontology Language (OWL) (and related languages like the Resource Description Framework Schema, RDF) became widely used (Bodenreider and Stevens, 2006). In this context, there is ongoing work on translating ApiNATOMY schema into RDF and OWL to enable integration of generated physiology models with SciCrunch (Grethe et al., 2014).

Directly describing input models of physiology routes in the ontological formats seems very in-practical and time-consuming. User-friendly versions of OWL such as Manchester syntax (W3C Working Group) make the textual inspection and editing of ontologies easier and could potentially be used by advanced modelers to specify physiology models. However, on the one hand, these languages focus on the level of detail not needed in our application. On the other hand, they are rather complex, not widely known, and require specialized expertise. Finally, we would need to translate such specifications into suitable format for data exchange and in-browser visualization.

For the latter reason, we opted for JSON as a format for user-level model definition. It is a generic data format that itself only defines how data is stored on a very basic level, it is simple and requires minimum overhead in processing. Among other essential technologies widely used in web applications are three data formats built on JSON: GraphQL (GraphQL Foundation), JSON-LD (W3C JSON-LD Working Group) [(JSON for Linking Data), and JSON Schema (IETF Working Group)]. GraphQL is a data query language that uses JSON both for data requests and responses. Using GraphQL, one can shape a form of JSON request, expecting to get data back in the exact same format. GraphQL is an alternative for a RESTful API and is used largely for data fetching in web applications. JSON-LD adds meaning to JSON documents. Using this data format, one can enrich the data in JSON documents with metadata reflecting the semantics of the content.

Graphical visualization remains an important enabler in many practical tasks related to bio-medical modeling. Numerous visualization methods for ontologies have been developed (Dudás et al., 2018). Most of these approaches are designed to display given structures and navigate the knowledge graphs, paying little attention to the process of acquisition and management of the data. Consequently, graphical modeling tools are rarely used for large-scale knowledge base populating or re-factoring. We tackle this issue with provision of the notion of modeling templates to instantly generate large sub-graphs. Modeling templates can also be used to encapsulate specialized formats for anatomical structure generation such as e.g., L-systems (Prusinkiewicz and Lindenmayer, 1990).

## 7. Conclusions

In this paper, we presented the KR model for the ApiNATOMY framework, a modeling methodology of multi-scale physiology systems. We described the key modeling classes and illustrated their use to model and visualize physiology structures.

While there are many well-structured formats to accurately capture the semantic relationships among organs, cells, proteins, etc., none of them are working across scale in a way that enables automated generation of visual route schematics reflecting entity interaction within a given context. With ApiNATOMY KR model and KM toolset, we are able to model and display structural information (e.g., show regional and constitutional parts of various compartments, define material composition), satisfy spatial constraints (i.e., position process edges within compartments or their borders), automatically generate resources to model complex assemblies (arborizations, membrane channels), associate resources with various data from ontological terms to computational models.

The ApiNATOMY KR has been designed to accommodate frequent changes and updates. Model authors working in certain areas of physiology may require modeling templates which allow them to quickly describe recurring patterns in the system under study. With the use of JSON for model definition and extended JSON Schema for generating entity-relationship diagrams, we succeeded to create a working prototype satisfying given system requirements.

## Data Availability Statement

Publicly available datasets were analyzed in this study. This data can be found here: https://github.com/open-physiology/open-physiology-viewer.

## Author Contributions

BB designed a conceptual framework for the topological and semantic assembly of multiscale physiology route maps. The framework, called ApiNATOMY, consists of a knowledge representation (KR) model and a set of knowledge management (KM) tools. All authors contributed to the article and approved the submitted version.

## Conflict of Interest

The authors declare that the research was conducted in the absence of any commercial or financial relationships that could be construed as a potential conflict of interest.
