# Erratum: Knowledge Representation for Multi-Scale Physiology Route Modeling

**DOI:** 10.3389/fninf.2022.930152

**Published:** 2022-05-16

**Authors:** Frontiers Production Office

**Affiliations:** Frontiers Media SA, Lausanne, Switzerland

**Keywords:** physiology, multi-scale model, knowledge management, anatomy, connectivity, ontology

Due to a production error, there was an error regarding the affiliations for author Bernard de Bono. Instead of affiliation 1, he should only be associated with affiliation 2 “Auckland Bioengineering Institute, University of Auckland, Auckland, New Zealand”.

The publisher apologizes for this mistake. The original version of this article has been updated.

